# CT-based methods for the assessment of glenoid bone loss

**DOI:** 10.1007/s00256-026-05357-2

**Published:** 2026-09-07

**Authors:** Massimo De Filippo, Michele Fischetti, Carlo Felice De Biase, Marcello Zappia, Giorgio Concari, Mario Brunese, Chiara Martini, Ruben Foresti, Lorenzo Bertolotti, Ganiyat Adenike Ralitsa Adebanjo, Giulia Viviano

**Affiliations:** 1 Present Address: Department of Medicine and Surgery, Section of Radiology, University of Parma, Maggiore Hospital, Via Gramsci 14, 43126 Parma, PR Italy; 2 Centro Diagnostico Ricerche Radiologiche S.R.L., Via Pierluigi da Palestrina 1, 70056 Molfetta, Italy; 3 Casa Di Cura Città Di Parma, P. Le Athos Maestri 5, 43123 Parma, Italy; 4 Department of Medicine and Health Science “V. Tiberio”, University of Molise, 86100 Campobasso, Italy; 5 Department of Precision Medicine, University of Campania “L. Vanvitelli”, 80138 Naples, Italy

**Keywords:** Glenoid bone loss, Shoulder instability, CT imaging, PICO method, PARMA method, On-track/off-track classification

## Abstract

Recurrent anterior shoulder dislocation often causes progressive damage and bone loss of the anteroinferior glenoid rim, which is a key factor in persistent shoulder instability. Correct measurement of bone loss is essential before surgery, as it helps decide whether a soft tissue repair is sufficient or if a bone graft procedure, such as the Latarjet, is required. In the past, arthroscopy was considered the standard method for evaluating glenoid bone loss, using the so-called “bare spot” as a reference point. However, this landmark is not always visible and has shown poor reliability, which has reduced its clinical value. For this reason, imaging methods with CT and MRI are now preferred. One of the best-known imaging techniques is the PICO CT method, which calculates bone loss by inscribing the glenoid in a circle. Although widely used, this method has significant limitations because the glenoid surface is not truly circular. As a result, variability between observers is high, and clinical accuracy is reduced. To improve precision, the PARMA method was developed. This technique uses curved multiplanar CT reconstructions (cMPRs) to follow the real concavity of the glenoid surface. Cadaveric studies have shown that PARMA is more accurate and reproducible than traditional CT methods, with good agreement compared to high-precision laser scanning. In addition, Hill–Sachs lesions must also be evaluated. The on-track/off-track concept combines humeral and glenoid bone loss to predict the risk of recurrent dislocation. Off-track lesions have a higher chance of surgical failure if treated with soft tissue repair alone and usually require bone procedures. Overall, modern CT-based techniques and combined lesion assessment improve accuracy and allow more tailored treatment of shoulder instability. The aim of this review is to provide an overview of the CT methods most employed to quantify glenoid bone loss in traumatic shoulder instability, emphasizing their strengths and limitations.

## Introduction

The shoulder is the joint of the body that most frequently undergoes dislocation, largely because of its wide range of motion, unmatched in humans. The particular anatomy of the shoulder (a ball-and-socket joint) contributes to this extensive mobility and, given the smaller articular surface of the glenoid (about one-third) compared with the humeral head (two-thirds of a sphere), there is a high risk of instability and dislocation. Between 1 and 2% of the population will experience a glenohumeral dislocation during their lifetime [1].

More than 95% of glenohumeral joint dislocations are anterior; consequently, the humeral head most commonly displaces in an anteroinferior direction, moving away from the shallow glenoid cavity [2]. Superior translation of the humeral head is limited and contained by the osteo–musculo–tendinous complex formed by the acromion, the coracoid process, and the rotator cuff.

The initial injury often occurs after a fall onto an outstretched arm or directly onto the shoulder. The resulting anatomical injuries may involve different regions either in isolation or simultaneously; in particular, injuries to the glenohumeral ligaments or the fibrocartilaginous glenoid labrum may be observed, as well as bony fractures of the glenoid and/or the humeral head and sometimes associated traumatic injuries of the rotator cuff. Many patients with anterior shoulder dislocation sustain a posterolateral impaction fracture of the humeral head [3]. A Hill–Sachs lesion appears as a focal indentation or concavity along the posterosuperolateral profile of the humeral head and represents an indicator of prior stability. Specifically, the greater the depth of the Hill–Sachs fracture, the greater the trauma responsible for the dislocation and, consequently, the greater the stability prior to the injury; conversely, when the Hill–Sachs fracture is absent or shallow, the trauma responsible for the dislocation is less severe and the shoulder was less stable beforehand. When these fractures occur simultaneously (in approximately four out of five dislocations [81%]), they are referred to as bipolar bone lesions, as they are almost diametrically opposite each other relative to the center of motion of the shoulder joint. Of all patients who sustain an anterior shoulder dislocation, 20–48% will experience recurrence, which is more common in younger individuals, with recurrence rates of 66–94% in patients younger than 20 years of age [4].

Recurrent anterior dislocation of the humeral head progressively leads to wear, remodeling, and bone loss of the anteroinferior rim of the glenoid [5]. The prevalence of glenoid bone loss in cases of traumatic unidirectional shoulder instability—therefore referred to as TUBS (traumatic, unidirectional, Bankart lesion, surgical)—varies widely, ranging from 8 to 90% depending on published series [6, 7].

Glenoid bone loss represents a significant risk factor for recurrent glenohumeral instability [8–11]. This may result from an acute fracture (bony Bankart lesion) or from progressive erosion of the anteroinferior glenoid rim secondary to repeated impacts between the humeral head and the glenoid margin, typically seen in patients with single or recurrent dislocations [5, 8]. Several studies have proposed critical thresholds of glenoid bone loss between 10 and 25% [12]. A recent consensus statement suggests considering bone graft reconstruction when glenoid defects exceed 20% [13].

Therefore, preoperative evaluation of glenoid bone loss is essential for surgical planning, particularly in deciding between arthroscopic capsulolabral repair and more invasive bone graft procedures such as Latarjet or other bone block techniques, either open or arthroscopic [5, 14]. The Latarjet–Putti procedure involves transposition of the coracoid process to the anteroinferior glenoid rim, below the glenoid equator, with fixation using metallic screws, thereby creating a bony and dynamic restraint against anterior humeral head translation.

Numerous methods have been proposed for evaluating the glenoid bone, the main ones being arthroscopic and noninvasive imaging. Among these, the most used is CT without contrast agent; MRI is currently less commonly used due to its low spatial resolution and nonspecificity for calcium.

## Arthroscopic method

**Fig. 1:**
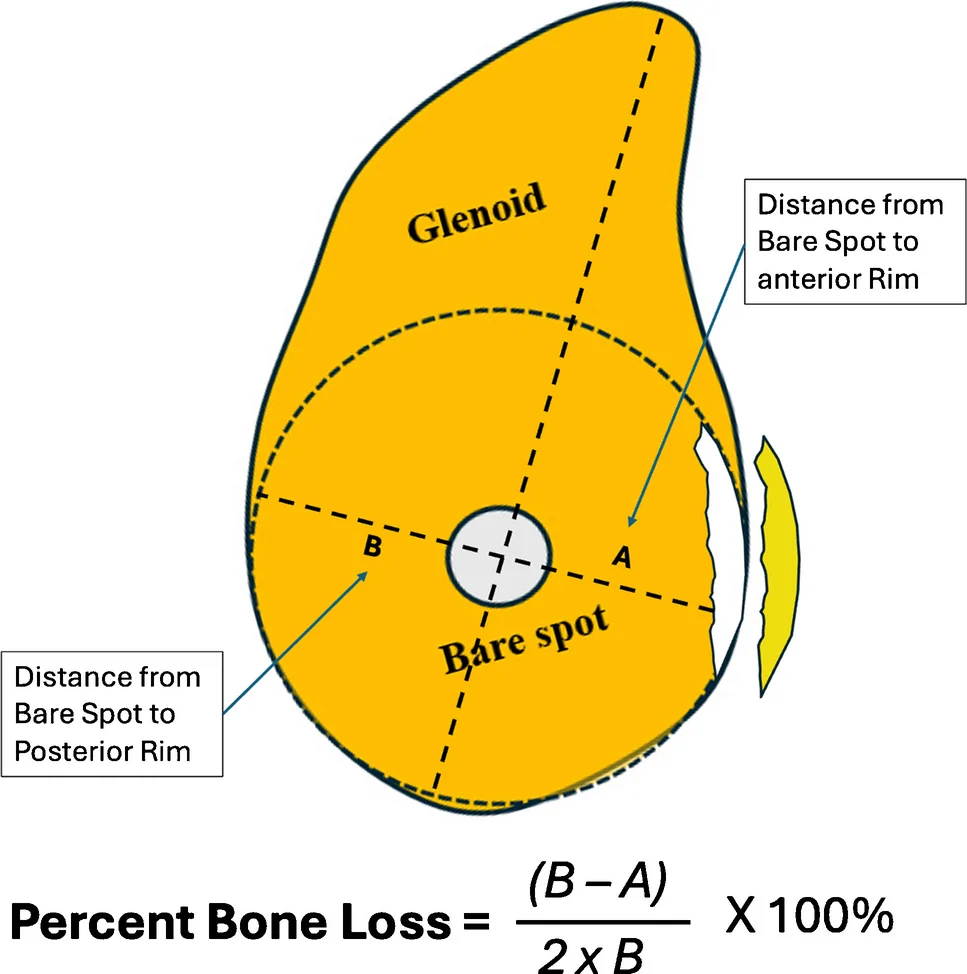
Arthroscopic method. The Burkhart bare spot method uses a 3-mm area of cartilage on the inferior glenoid, known as the glenoid bare spot (GBS), as a consistent landmark to arthroscopically measure and quantify glenoid bone loss in patients with anterior shoulder instability. The distance from the GBS to the posterior and intact anterior glenoid rims is measured, with a shorter anterior distance indicating bone loss. However, the validity of this method has been questioned due to the inconsistent presence and central location of the GBS in some patients, leading to potential inaccuracies in bone loss measurements

Traditionally, arthroscopic evaluation of glenoid bone loss has been considered the reference standard for quantification [15]. As described by Burkhart et al. [15], this method is based on identifying the therefore-called bare spot, a cartilage-free area located approximately at the center of the circle that can be drawn along the inferior glenoid rim. The bare spot is used as a reference point to estimate bone loss (Fig. 1).

However, some authors [16] have questioned the reliability of the bare spot as an anatomical landmark. It has been observed that this structure is visible in less than 48% of patients undergoing shoulder arthroscopy and in less than 26% of patients assessed with CT arthrography. Furthermore, anatomical studies have demonstrated that the bare spot is not precisely centered in the inferior glenoid [17–19], and its use may lead to overestimation of small defects [20]. Given these limitations, arthroscopy can no longer be considered a true reference standard for glenoid bone loss quantification. Over time, less-invasive radiological methods have been introduced, particularly CT- and MRI-based techniques. In this context, an Italian study compared different measurement techniques performed by CT and magnetic resonance imaging (MRI), highlighting that the PICO method on CT, among the methods considered, is the one that provides the best results since it provides a relatively low intra- and interobserver variability; however, this method cannot be considered reliable, as the study is strongly limited by a bias related to the absence of a reference standard [21].

## PICO method

**Fig. 2:**
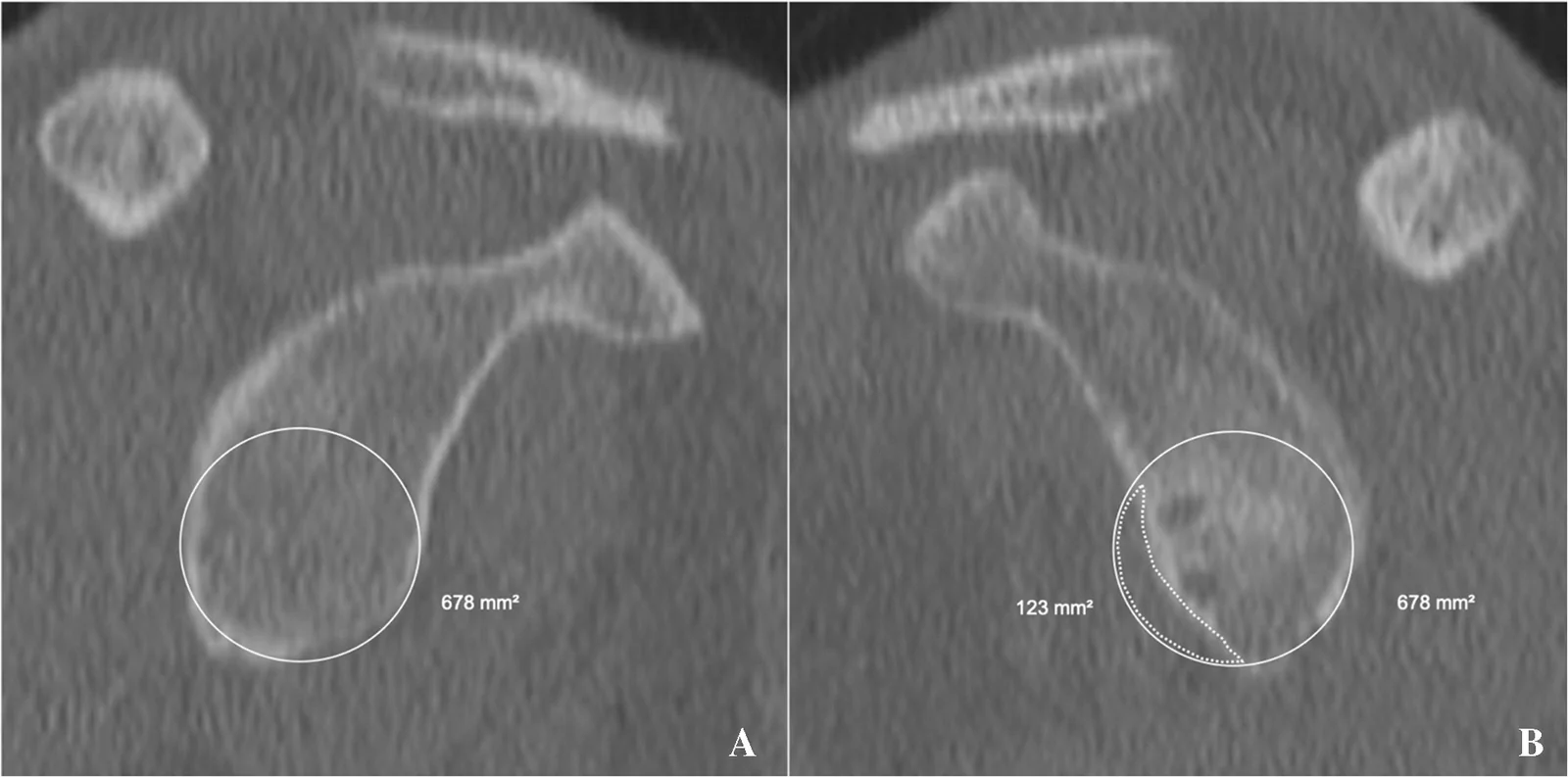
PICO method. Quantification of glenoid bone loss on 2D CT with multiplanar reconstructions (MPRs), with the glenoid viewed en face. The inferior scapular glenoid of the healthy shoulder is inscribed in a circle (**A**). The software then calculates the delimited area of the border. The circle is translated onto the unstable contralateral glenoid. The area of bone loss (in this case 123 mm^2^) is manually delineated (**B**). Finally, the area of the unstable glenoid is subtracted from the area of the healthy glenoid and the percentage of bone loss between the two glenoids is calculated

The PICO CT method, first proposed by Baudi in 2005, is based on oblique sagittal sections of the glenoid with a thickness ranging from 0.6 to 1.0 mm, obtained as a result of bidimensional multiplanar reconstructions (MPRs). The glenoid surface is calculated using a circular region of interest (ROI) traced along the inferior margin of the healthy glenoid. The same circle is then superimposed onto the reconstructed sagittal image of the affected glenoid, and the missing surface is manually defined. The difference between the two surfaces allows calculation of bone loss percentage by comparing with the intact contralateral glenoid [22] (Fig. 2).

**Fig. 3:**
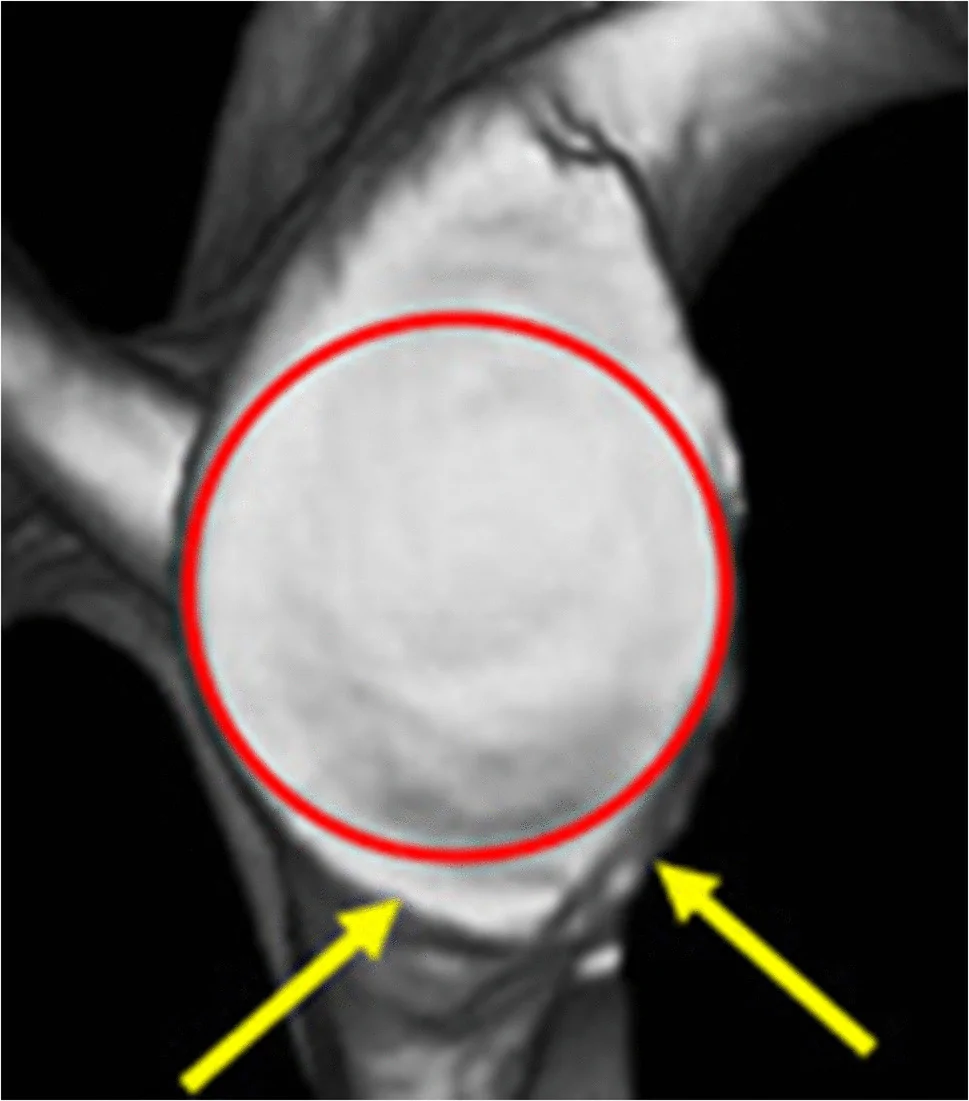
3D CT reconstruction of the scapular glenoid in the sagittal plane. The region of interest of the lower glenoid in the circle does not correspond to the real anatomy of the glenoid surface as it lacks the bone surface inferior to the circle (arrows, 6 o’clock)

In particular, the main weakness of the PICO CT method lies in its two-dimensional geometric approach, which may represent a limitation in the evaluation of surfaces with intrinsic anatomical and post-traumatic variability; indeed, fitting the inferior glenoid into a circle on 2D CT images may not accurately represent the true anatomy of the glenoid surface, which is not only curved but also morphologically difficult to approximate as a circle (Fig. 3) [23].

Furthermore, this circle is created only if the sagittal scanning plane is immediately below the articular plane (if you are 1–2 mm below the glenoid, the circle is created; if you go up in the osteochondral plane, it is never a circle). Anatomical findings are highly subjective and variable; different inclination of 2 or 3° between observers reduces or increases the diameter (2 or 3 mm) with consequent significant variability of the surface (about 30%); sometimes there is bone surface below the circle (6 o’clock) (Fig. 3).

De Filippo et al. [23] tested the reproducibility of the PICO CT method in a cadaveric model by comparing these measurements with those obtained by a high-precision laser probe for optical scanning. It emerged that the interobserver variability for the CT measurement of the glenoid surface using the PICO CT method was high compared to the laser, in the evaluation of the glenoid surface in cadaveric samples; therefore, the PICO CT method is not reliable and may cause errors in the clinical management of the patient.

## PARMA method

To overcome the limitations of the PICO method, De Filippo et al. developed the PARMA method, a noninvasive and reproducible technique to assess glenoid bone loss by CT with multiplanar “curved” reconstructions (cMPRs), using a cadaveric model [24].

**Fig. 4:**
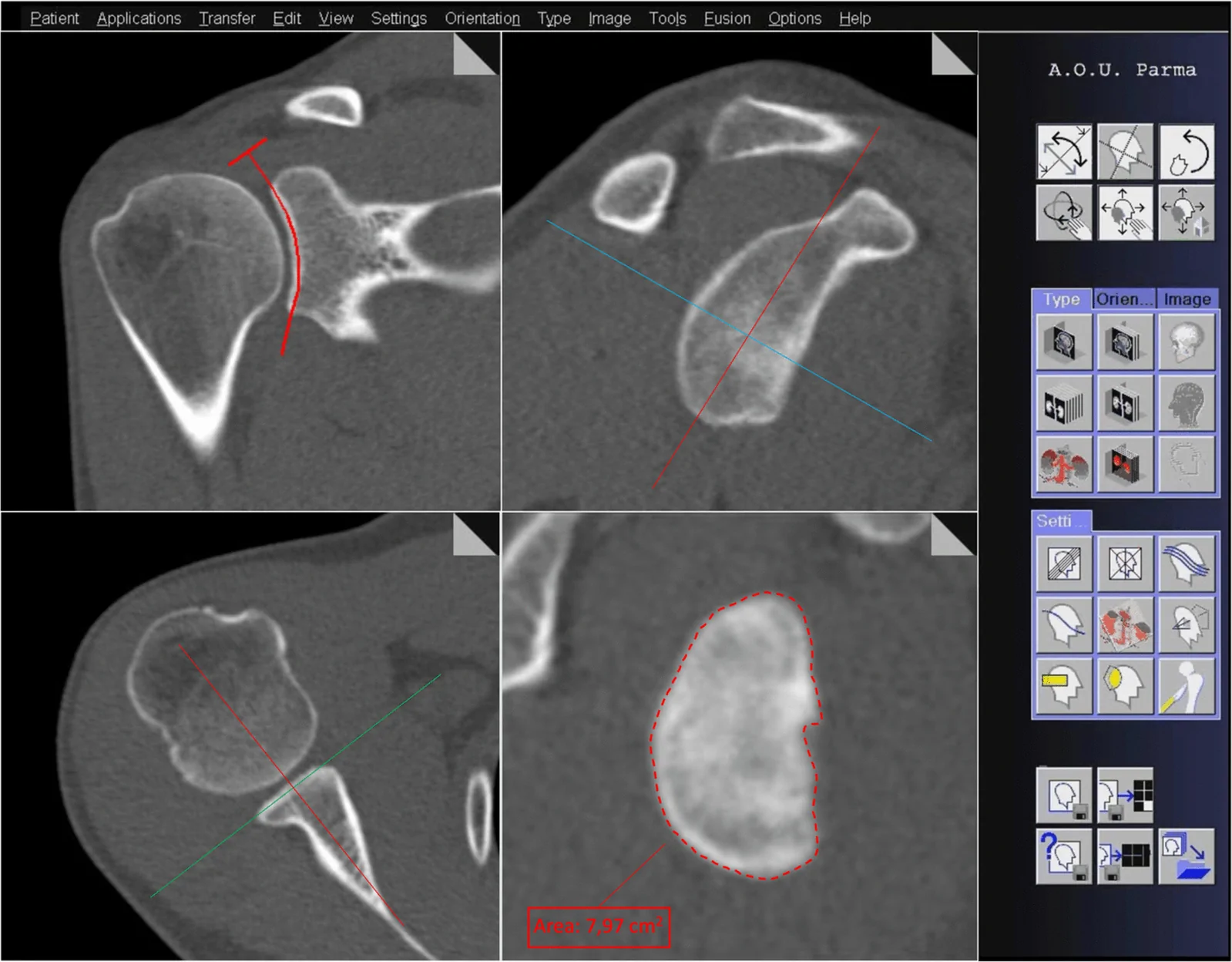
PARMA method. Radiological workflow using cMPR for glenoid surface assessment: top-right and bottom-left images display the orientation of sagittal and axial planes to align with the glenoid articular surface. Top-left image shows the resulting coronal view, where a curved plane tailored to the glenoid’s concavity is made. The result of this curved cut is depicted in the bottom right, where an “en face” view of the glenoid surface is presented. A hand-drawn region of interest (ROI—dotted red) is marked along the glenoid’s border, allowing for precise measurements of its total area. Finally, the area of the intact contralateral glenoid is measured using the same method; the area of the unstable glenoid is subtracted from the area of the healthy glenoid, and the percentage of bone loss between the two glenoids is calculated

To validate the accuracy and reproducibility of the cMPR technique, ten dry cadaveric scapulae were used, two of which were modified to simulate a Bankart-type bony lesion. Each specimen underwent CT scanning, followed by MPR in two modes: the traditional “flat” (MPR) and the “curved” (cMPR) (Fig. 4).

The cMPR technique allows following the natural concavity of the glenoid articular surface, generating curved reconstruction planes that are more adherent to the real anatomy of the glenoid than the images obtained with classic plane reconstructions. In both cases, the glenoid joint area was measured by manually tracing a contour (region of interest, ROI) along the outer margins. As an accuracy reference, a high-precision laser optical scanning system (CAM2 Laser Line Probe) was used, capable of generating a three-dimensional point cloud of the bone surface. The data collected was processed with 3D modeling therefore a software to obtain exact measurements of the area. Three experienced radiologists performed the CT measurements independently, and one of them repeated the analysis 3 months later to assess intraoperative reproducibility. The setting of the cMPR plane was performed by identifying the concave axis of the glenoid articular surface, tracing a curve in the oblique coronal plane. Finally, the results obtained with the MPR and cMPR techniques were compared with the laser measurements in terms of accuracy and variability.

The use of cMPR CT of the glenoid in a cadaveric model proved to be reproducible and significantly more accurate compared to conventional MPR. Moreover, cMPR CT provides reliable information despite the considerable anatomical variability of the glenoid surface [24]. As with the PICO method, the PARMA method is based on comparison with the contralateral glenoid. Both shoulders are included in a single CT acquisition; no additional CT scan and therefore no further radiation exposure are required.

## On-track/off-track

The concept of “on-track/off-track” arises from the combination of Hill–Sachs injury and glenoid bone loss.

**Fig. 5:**
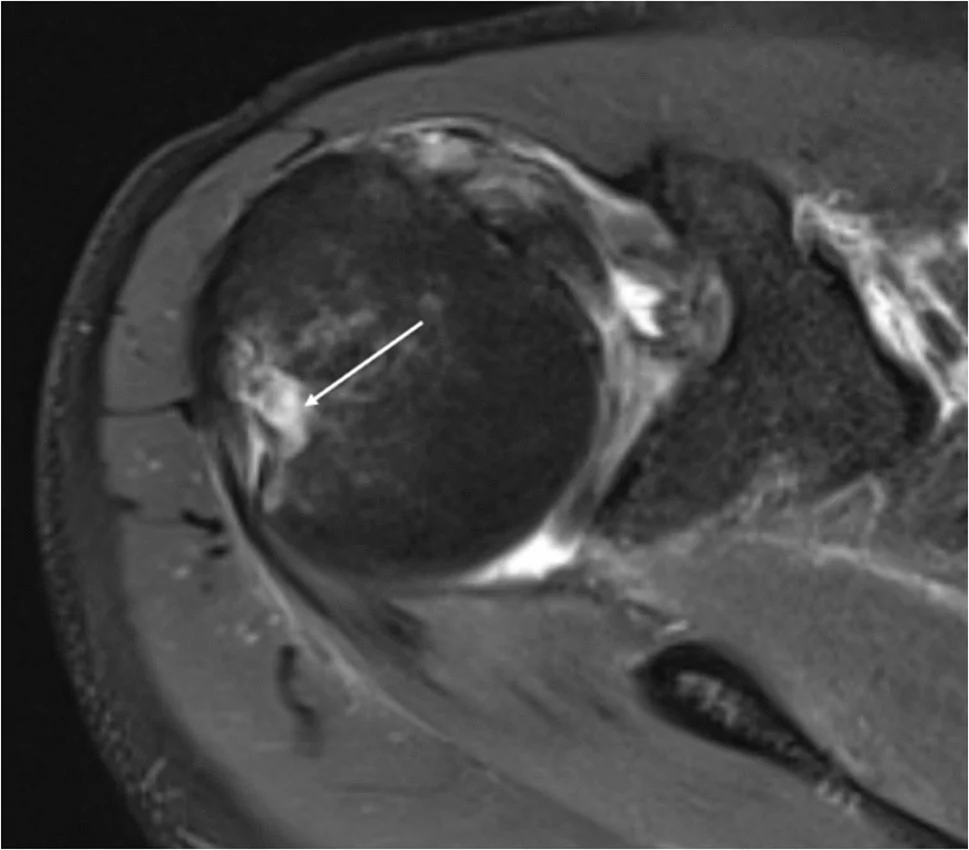
Hill–Sachs lesion. Depression of the posterior-supero-lateral cortical of the humeral head that occurs as a result of an anterior shoulder dislocation (arrow)

Hill–Sachs injury is a depression of the posterior-supero-lateral cortical of the humeral head that occurs because of an anterior shoulder dislocation (Fig. 5). This injury is particularly relevant in the evaluation of glenohumeral instability, especially in patients who have had recurrent episodes of dislocation.

The idea is that not all Hill–Sachs lesions are clinically significant: They become problematic only when they are large enough and oriented to contact the anterior margin of the glenoid during the abduction and extrarotation movement, causing incarceration subsequently a new dislocation. This potentially pathological contact is termed “off-track,” while a lesion that remains “inside the track,” and therefore does not conflict with the glenoid margin, is considered “on-track” [25, 26]. To assess shoulder stability in the presence of bipolar bone loss involving both the glenoid and the humeral head, Yamamoto et al. proposed the concept of the “glenoid track.”

**Fig. 6:**
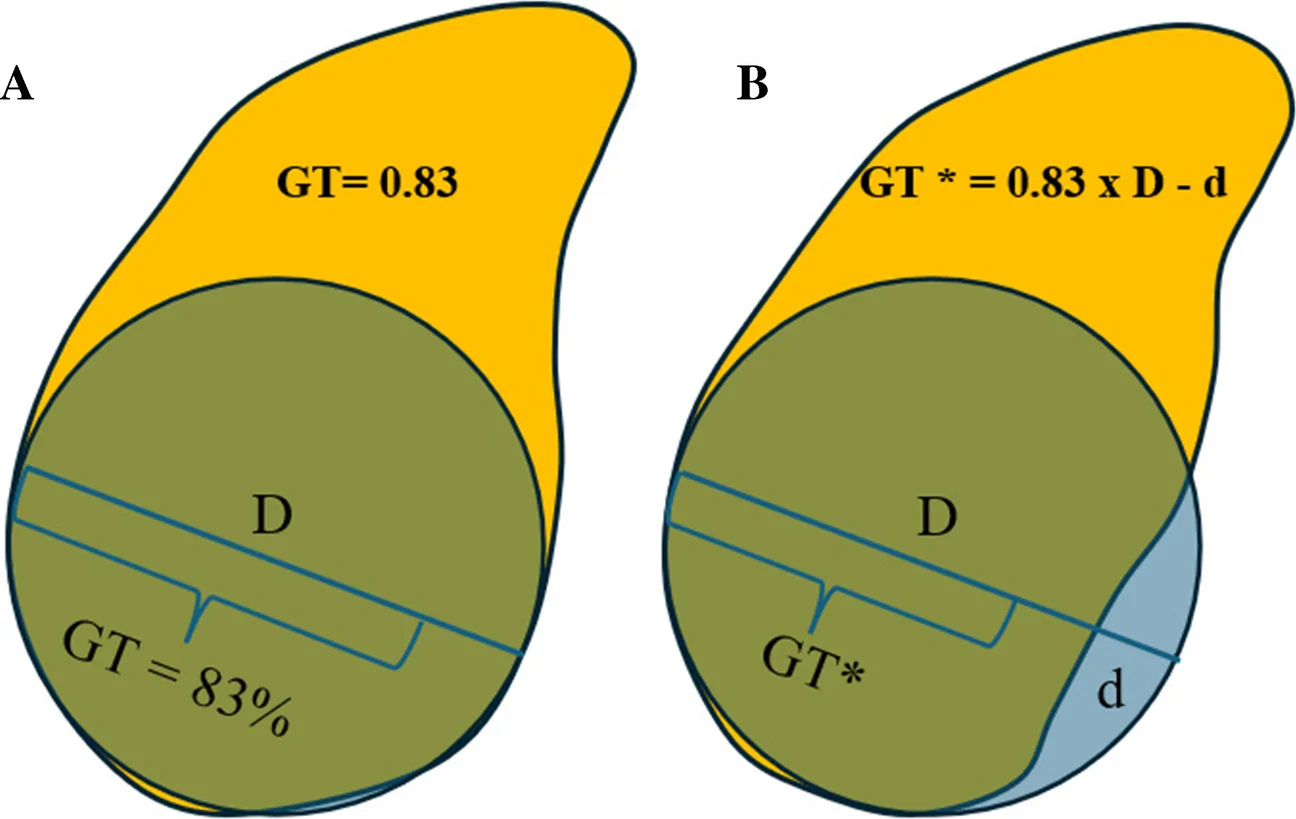
Glenoid track. **A** In the absence of glenoid defects, the glenoid track is 83% of the glenoid width. **B** In the presence of a bone defect at the anterior edge of the glenoid, the width of the defect (*d*) must be subtracted from 83% of the length to obtain the true width of the glenoid pathway (GT*)

The calculation of the “track” is based on the analysis of the concept of “glenoid track” (GT), which represents the area of the humeral head that normally articulates with the glenoid during the abduction and external rotation movement. Under normal conditions, the glenoid track is about 83% of the width of the glenoid (Fig. 6A).

When there is an anterior bone loss of the glenoid, the width of the track is reduced (Fig. 6B).

**Fig. 7:**
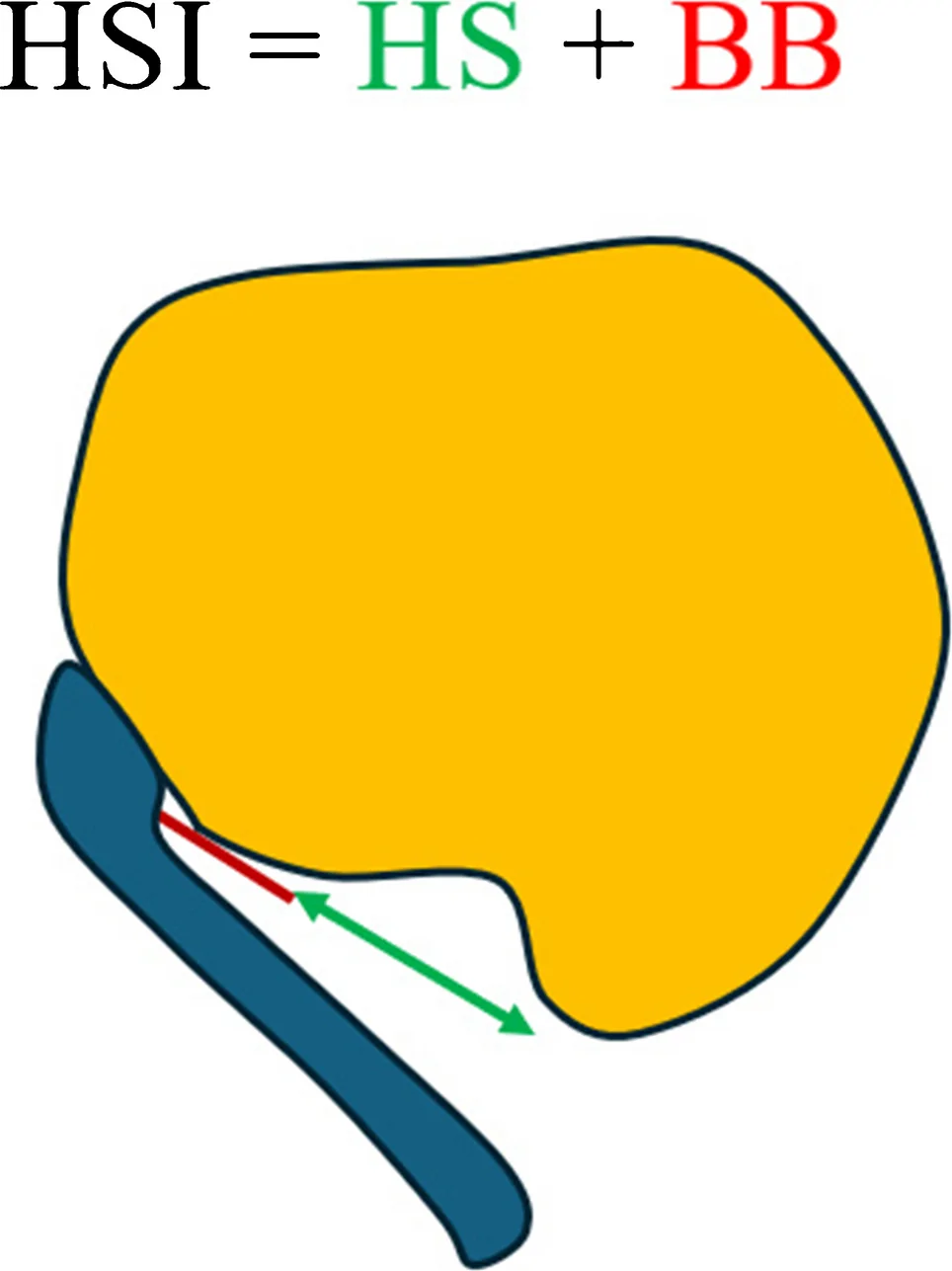
Hill–Sachs interval (HSI). The green line represents the Hill–Sachs defect (HS); the red line represents the bone bridge (BB) between the defect and the cuff insertion. These lines are summed to characterize the Hill–Sachs interval used in the glenoid track calculation

Next, the Hill–Sachs interval (HSI) is calculated (Fig. 7), defined as the sum of the Hill–Sachs lesion and the bone bridge between the defect and the cuff insertion.

Measurement by imaging, especially with CT and MRI, is critical to classify lesions. After calculating the true glenoid track (GT*), which accounts for anterior glenoid bone loss (GT* = 0.83 × *D* − *d*), the Hill–Sachs interval (HSI) is compared with GT*.

HSI < GT* = Hill–Sachs “ON TRACK” (nonengaging).

HSI > GT* = Hill–Sachs “OFF TRACK” (engaging) [27, 28].

The clinical value of this classification lies in its predictive capacity: Patients with off-track lesions have a much higher risk of post-surgical recurrence if treated only with tissue stabilization surgery (such as Bankart). Additional bone treatment, such as the Latarjet procedure or *remplissage*, may be indicated in these cases [5].

## Discussion

Anterior shoulder dislocation is a common injury, particularly among young patients and athletes, and is often associated with a high recurrence rate.

Multiple factors are considered when determining the treatment strategy for anterior shoulder instability, including patient age and sex, the extent of glenoid bone loss, bipolar bone loss (glenoid bone loss combined with a Hill–Sachs lesion), hand dominance, athletic status, number of previous dislocations, prior shoulder surgery, generalized hyperlaxity, patient expectations, and the ability to comply with postoperative rehabilitation [29].

The integrity of the glenoid bone plays a pivotal role in maintaining shoulder stability, with the risk of recurrent dislocation being closely related to the magnitude of bone loss. Surgeons frequently consider both the degree and location of glenoid bone loss when choosing between soft tissue and bony stabilization procedures. This highlights the importance of accurately measuring bone loss to guide therapeutic decision-making and underscores the need for reliable assessment methods.

In 2000, arthroscopy was proposed as a reference method for calculating glenoid bone defects (Burkhart et al.), as it allows direct visualization of the glenoid surface. However, its utility for precise quantification of bone loss is limited. Although arthroscopy enables in vivo measurements of the glenoid, it is invasive, and given that the primary goal of surgical planning is measurement acquisition, a noninvasive approach would be preferable. Moreover, arthroscopic assessment is essentially two-dimensional and relies on linear diameters, which may be less accurate than surface-based measurements. In addition, this quantification depends on the presence of the bare spot as an anatomical reference for the glenoid center, which may be absent and whose position is highly variable. Therefore, due to its invasiveness and limitations in providing accurate measurements, arthroscopy cannot be considered a true gold standard in this context.

To calculate the extent of bone loss, several imaging techniques have been proposed, including two-dimensional (2D) and three-dimensional (3D) computed tomography (CT) and magnetic resonance imaging (MRI). Various measurement methods have also been developed, including linear and surface-based approaches. Linear methods primarily involve length and diameter measurements, whereas surface-based techniques focus on quantifying the area of bone loss. Among these, the PICO method is the most widely used because it is relatively accurate and simple to apply, and it is commonly employed regardless of the imaging modality.

As described by Baudi et al., the PICO method uses multiplanar reconstruction (MPR) to obtain an en face view of the glenoid. A circle is drawn on the inferior portion of the intact glenoid and then transferred to the contralateral side, where the area of bone loss is outlined.

However, inscribing the inferior glenoid within a circle does not reflect the true anatomy of the glenoid surface, which is difficult to approximate with a circular shape. Consequently, portions of the inferior glenoid surface may not be included, even though abnormalities of the inferior glenoid surface have been reported in up to 90% of TUBS shoulders, thereby reducing the accuracy of the PICO method in estimating true bone loss. This limitation becomes particularly relevant when bone loss is substantial, as the defect may extend beyond the perimeter defined by the PICO circle.

For the same reason, drawing a circle that best fits the inferior glenoid is subject to positional variability, since the anatomical landmarks required for circle placement are not clearly defined. This variability affects the radius length and, consequently, the calculated area. Furthermore, studies using the PICO method—whether based on planar MPR or 3D volumetric rendering—do not account for the concave nature of the glenoid when calculating its surface.

This planar assessment neglects the critical concavity of the glenoid, which is not only anatomically evident but also plays a fundamental role in shoulder stabilization. Ignoring this feature may lead to inaccuracies in evaluating glenoid morphology and bone loss, as the true three-dimensional structure of the glenoid is not adequately represented.

Compared with methods based on the best-fit circle, the curved multiplanar reconstruction (cMPR) method aims to overcome these inaccuracies and limitations. The cMPR technique incorporates a curved section plane to accurately account for the concave nature of the glenoid, ensuring precise measurement of the actual articular surface involved in the glenoid defect. This approach not only describes the true extent of bone loss but also eliminates subjectivity in circle placement by using the clearly identifiable glenoid perimeter as an anatomical reference, allowing measurement of the entire glenoid surface. Unlike circle-based methods, whole-surface measurement also includes bone defects involving the superior equatorial region and the inferior glenoid surface.

Finally, the aforementioned linear and surface-based methods, including the PICO technique, have not been validated against a true reference standard, whereas the accuracy of our method was validated using a 3D-printed glenoid, considered a gold standard. Consequently, the cMPR method provides a comprehensive, objective, and reproducible assessment of glenoid bone loss.

Bipolar bone lesions, in the form of fractures of the anterior glenoid rim and the posterolateral humeral head, are frequently associated with anterior shoulder dislocation and may represent either a cause or a consequence of recurrent instability. The evaluation of the glenoid track is an evolving concept that incorporates the pathomechanics of anterior shoulder instability into its management. Currently widely accepted by orthopedic surgeons, this concept has important implications for prognosis, treatment planning, and outcome assessment in anterior shoulder dislocation.

The glenoid track represents the contact zone between the humeral head and the glenoid during shoulder motion from neutral position to abduction and external rotation. Two key factors determine whether a Hill–Sachs lesion is classified as on-track or off-track: the glenoid track width (GTW) and the Hill–Sachs interval (HSI). If the GTW is smaller than the HSI, the HSL is considered off-track; if the GTW exceeds the HSI, the lesion is considered on-track.

The authors focus on the rationale underlying the glenoid track concept and describe the stepwise assessment of the glenoid track using CT or MRI. Conversion from an off-track to an on-track lesion is a primary objective in the surgical stabilization of anterior shoulder instability. The pivotal role of imaging in glenoid track evaluation justifies the need for radiologists to be familiar with this concept, its challenges, and potential pitfalls and to produce clinically meaningful and actionable radiology reports for orthopedic surgeons, ultimately benefiting patient care.

## Final consideration

In conclusion, among the CT-based methods described for assessing glenoid bone loss, the PARMA method and the on-track/off-track classification appear to be the most accurate. They represent a significant advancement in the preoperative planning of shoulder instability, providing dynamic integration of bony anatomy into clinical decision-making.

## Data Availability

Data sharing not applicable to this article as no datasets were generated or analyzed during the current study.

## Abbreviations

TUBS: Traumatic, unidirectional, Bankart lesion, surgical
CT: Computed tomography
MRI: Magnetic resonance imaging
MPR: Multiplanar reconstruction
ROI: Region of interest
cMPR: Curved multiplanar reconstruction
HS: Hill–Sachs
HSI: Hill–Sachs interval
GT: Glenoid track
